# Deep learning increases the availability of organism photographs taken by citizens in citizen science programs

**DOI:** 10.1038/s41598-022-05163-5

**Published:** 2022-01-24

**Authors:** Yukari Suzuki-Ohno, Thomas Westfechtel, Jun Yokoyama, Kazunori Ohno, Tohru Nakashizuka, Masakado Kawata, Takayuki Okatani

**Affiliations:** 1 Graduate School of Life Sciences, Tohoku University, 6-3 Aoba, Aramaki-aza, Aoba-ku, Sendai, Miyagi 980-8578 Japan; 2 Department of System Information Sciences, Graduate School of Information Sciences, Tohoku University, 6-6-01 Aoba, Aramaki-aza, Aoba-ku, Sendai, Miyagi 980-8579 Japan; 3 Faculty of Science, Yamagata University, 1-4-12 Kojirakawa, Yamagata, Yamagata 990-8560 Japan; 4 New Industry Creation Hatchery Center, Tohoku University, 468-1 Aoba, Aramaki-aza, Aoba-ku, Sendai, Miyagi 980-0845 Japan; 5 Research Institute for Humanity and Nature, Kamigamo-Motoyama 457-4, Kita-ku, Kyoto, 603-8047 Japan; 6 Present Address: Research Center for Advanced Science and Technology, The University of Tokyo, 4-6-1, Komaba, Meguro-ku, Tokyo, 153-8904 Japan; 7 Present Address: Forestry and Forest Products Research Institute, 1 Matsunosato, Tsukuba, Ibaraki 305-8687 Japan

**Keywords:** Ecology, Biodiversity

## Abstract

Citizen science programs using organism photographs have become popular, but there are two problems related to photographs. One problem is the low quality of photographs. It is laborious to identify species in photographs taken outdoors because they are out of focus, partially invisible, or under different lighting conditions. The other is difficulty for non-experts to identify species. Organisms usually have interspecific similarity and intraspecific variation, which hinder species identification by non-experts. Deep learning solves these problems and increases the availability of organism photographs. We trained a deep convolutional neural network, Xception, to identify bee species using various quality of bee photographs that were taken by citizens. These bees belonged to two honey bee species and 10 bumble bee species with interspecific similarity and intraspecific variation. We investigated the accuracy of species identification by biologists and deep learning. The accuracy of species identification by Xception (83.4%) was much higher than that of biologists (53.7%). When we grouped bee photographs by different colors resulting from intraspecific variation in addition to species, the accuracy of species identification by Xception increased to 84.7%. The collaboration with deep learning and experts will increase the reliability of species identification and their use for scientific researches.

## Introduction

The number of scientific researches using organism photographs has been increasing in the field of ecology^1–7^. Advances in digital cameras and photograph sharing via the internet have made it possible to obtain individual-level data on organisms from photographs without taking photographs ourselves. To collect organism photographs, researchers often use citizen science programs. Photographs collected in citizen science programs have been used to investigate or estimate species distributions^5,7–10^. These studies showed that photographs collected from citizen science programs have a potential to be used in scientific studies, especially in the fields of conservation ecology and invasion ecology.

**Figure 1:**
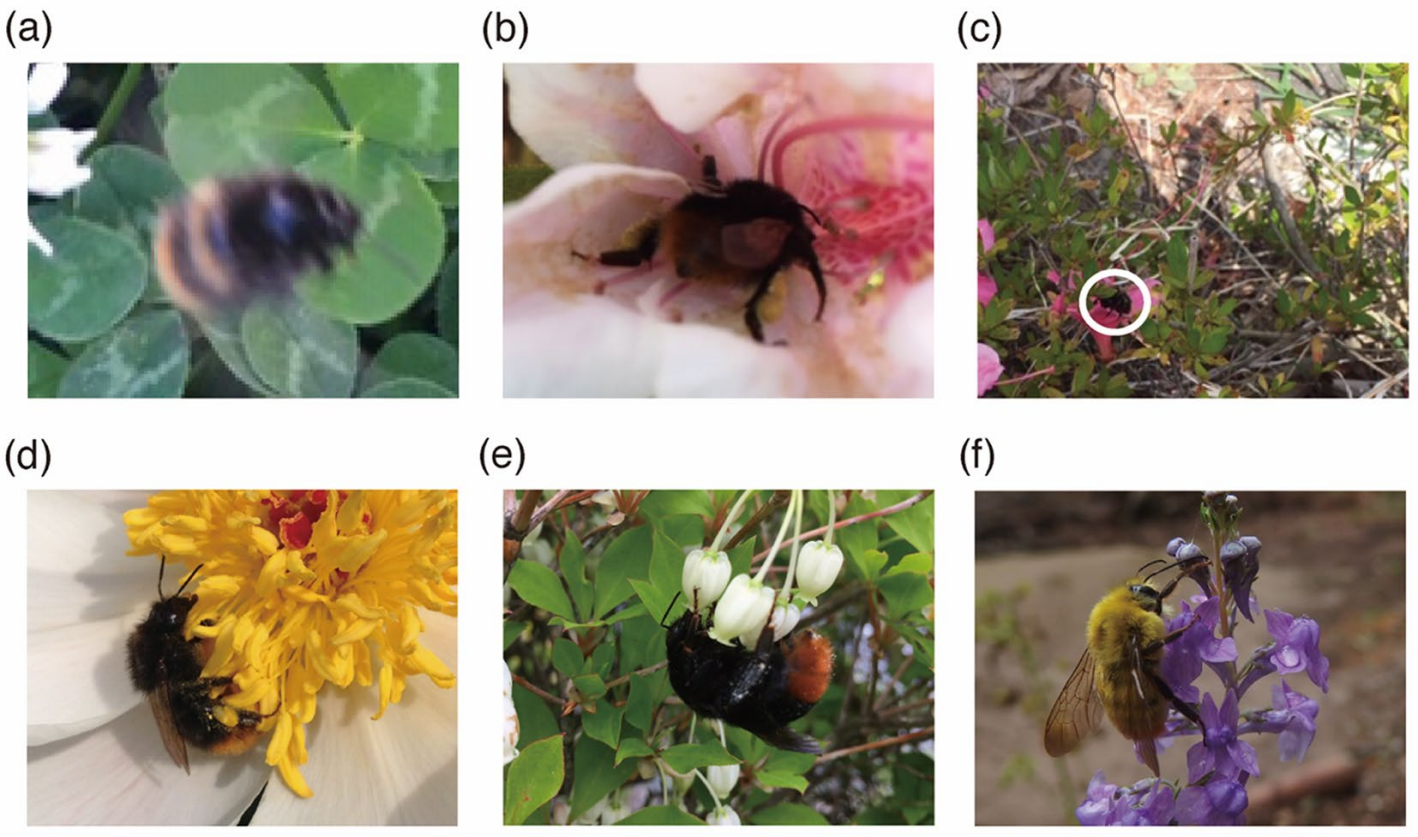
Difficulties of species identification of bees in photographs taken by citizens. Uppers represent low quality photographs. (**a**) Out of focus, (**b**) partially invisible, and (**c**) hard to see by shadow (a bumble bee appears within a white circle). Lowers represent interspecific similarity and intraspecific variation of bees. (**d**) Female *Bombus ardens ardens*, (**e**) female *B. ignitus*, and (**f**) male *B. ardens*.

While citizen science programs using organism photographs have become popular, there are two big problems related to photographs in citizen science. One problem is the low quality of photographs. When photographs are collected through citizen science programs, it sometimes results in low-quality photographs because animals move or hide under various lighting conditions outdoors (Fig. 1a–c). It increases the labor of identifying species in photographs. The other problem is the difficulty of species identification by non-experts due to interspecific similarity and intraspecific variation. Citizen science programs sometimes aim to monitor a specific group of organisms for conservation, and collect photographs of species in the same genus. Species in the same genus often have strong interspecific similarity, which produces less interspecific variation than intraspecific variation (Fig. 1d–f). These similarity and difference hinder species identification by human volunteers^11–14^. In citizen science programs “Blooms for Bees” and “BeeWatch”, the total accuracies of species identification by participants were 44% and 49%, respectively^14^.

To solve these problems, deep learning was selected to identify species in photographs taken by citizens. After the development of deep learning, the accuracy of object classification by convolutional neural networks (DCNNs) improved dramatically. DCNNs have succeeded in automatically identifying animals based on animal photographs captured by motion-sensor cameras in Serengeti National Park, Tanzania^15^. These photographs included partially invisible, too close, or far away animals with shadows under different lighting conditions, but DCNN can identify animals at 93.8% vs. experts’ identification. On the other hand, the accuracy of animal identification by human volunteers was 96.6% vs. experts’ identification. The difficulty of species identification by humans due to interspecific similarity and intraspecific variation was low in these animal photographs. It was expected that deep learning would be more useful for us when species identification was difficult due to interspecific similarity and intraspecific variation of target organisms. In citizen science programs using photographs, participants may not always be able to obtain the cooperation of experts in species identification. If the accuracy of species identification by deep learning is high, it will increase the availability and reliability of organism photographs collected in such citizen science programs, and enhance their use for scientific researches.

The aim of this study is investigating the accuracy of species identification by deep learning using various quality photographs of bee species with interspecific similarity and intraspecific variation. The photograph dataset used in this study consisted of 3779 photographs of two honey bee species and 10 bumble bee species in citizen science program “*Hanamaru-maruhana* national census (Bumble bee national census in English)”^8^. Of course, there is interspecific similarity within the same genus, but one of the major causes of interspecific similarity for these bee species is Müllerian mimicry^16,17^. Black body with orange tail like *B. ardens ardens* and *B. ignitus* (Fig. 1d,e) was considered as Müllerian mimicry^17,18^. The major causes of intraspecific variation are castes and phenotypic variation. Bumble bees have the castes of queen, worker, and male, and there are obvious differences between castes at least in body size and sometimes in hair color. The hair color of *B. ardens ardens* shows clear sexual dimorphism: black body with orange tail in females vs. yellow body with orange tail in males (Fig. 1d,f). Even in the same caste (especially worker), body size and color are different among individuals based on growing situations and ages^19^. For example, the hair color of *B. diversus* becomes yellowish when they inhabit colder regions, and their hair color becomes dull as they were older. In the special case of intraspecific variation, two subspecies of *B. ardens* have different hair colors from the nominotypical subspecies *B. ardens ardens*.

For defining the degree of interspecific similarity and intraspecific variation for humans, 50 people were tested to identify species using the photograph dataset. In the species identification test by humans, there is a risk of low accuracy regardless of interspecific similarity and intraspecific variation due to the difficulty of species identification itself. To see misidentification patterns by interspecific similarity and intraspecific variation, “biologists”, an intermediate group between the general participants and the expert identifying bee species in the citizen science program, were selected as subjects of the species identification test. They identified species of nine bee photographs selected randomly from the photograph dataset. The accuracy of species identification by biologists was also used to evaluate the accuracy of species identification by deep learning. Then, the accuracy of species identification by deep learning was investigated using the photograph dataset. A deep convolutional neural network, Xception^20^, was selected, and transfer learning^21,22^ and data augmentation^23^ were adopted to solve the issue of a shortage of photographs (Appendix S1 in Supplementary information). Xception learned species classes of the photograph dataset that were classified into different classes depending on bee species. Xception also learned color classes of the photograph dataset that were classified into different classes depending on color differences resulting from intraspecific variation in addition to bee species. The accuracies of identifying species classes and color classes by Xception were compared with that of identifying species classes by biologists.

## Results

### Species identification by biologists

To define the degree of interspecific similarity and intraspecific variation for humans and the accuracy of species identification by humans, we asked 50 biologists with different levels of knowledge to identify species using bee photographs. We calculated total accuracy, precision, recall, and F-score based on their answers. Precision is the proportion of correct answers for all answers of the target species. Recall is the proportion of correct answers for the target species. F-score is the harmonic average of the precision and recall.

**Figure 2:**
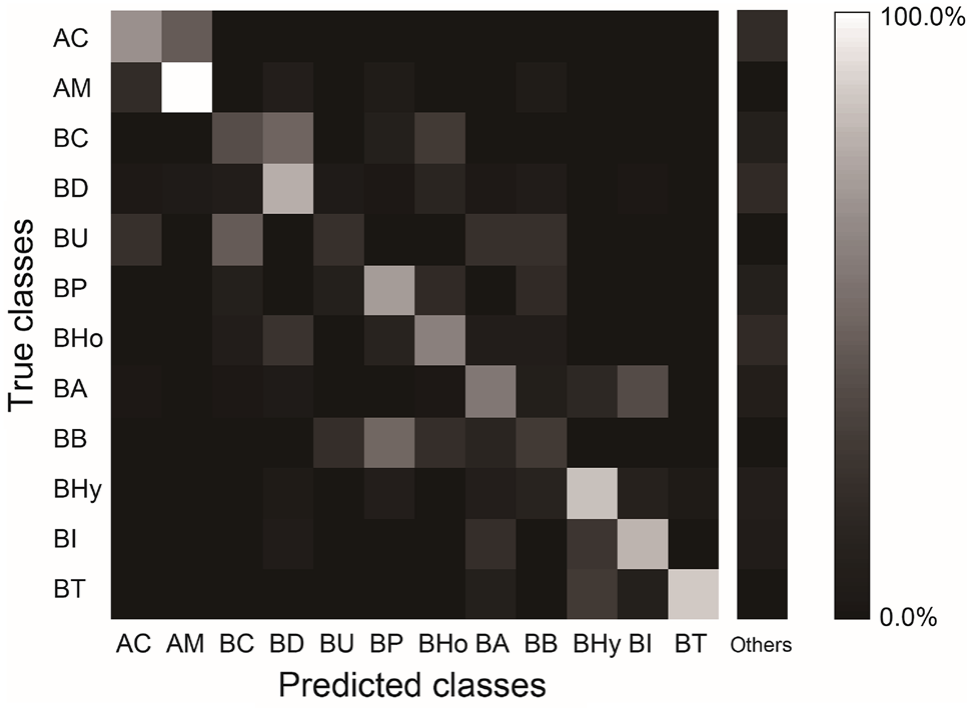
Confusion matrix by biologists. Bright color indicates high percentage of predicted classes for a true class. AC: *Apis cerana*, AM: *A. mellifera*, BC: *Bombus consobrinus*, BD: *B. diversus*, BU: *B. ussurensis*, BP: *B. pseudobaicalensis* and *B. deuteronymus*, BHo: *B. honshuensis*, BA: *B. ardens*, BB: *B. beaticola*, BHy: *B. hypocrita*, BI: *B. ignitus*, and BT: *B. terrestris*. Others means NA or the others.

The total accuracy of species identification by biologists was 53.7% vs. expert’s identification. Interspecific similarity between bumble bee species strongly affected the accuracy of species identification (Fig. 2). Interspecific similarity between female *B. ardens ardens* (black body with orange tail in Fig. 1d) and female *B. ignitus* (black body with orange tail in Fig. 1e) reduced the accuracy of species identification. Female *B. ardens ardens* was frequently misidentified as female *B. ignitus*, and female *B. ignitus* was also misidentified as *B. ardens* (Fig. 2). In addition, male *B. ignitus* (yellow body with black bands and orange tail) was often misidentified as male *B. hypocrita* (yellow body with black bands and orange tail) or female *B. hypocrita* (black body with cream bands and orange tail) (Fig. 2). It resulted in 37.7% precision and 57.1% recall of *B. ignitus*, and 44.2% recall of *B. ardens* (Table 1). Interspecific similarity between honey bee species also affected the accuracy of species identification. Native honey bee species *A. cerana* was often misidentified as domestic *A. mellifera*, and *A. mellifera* was also misidentified as *A. cerana* (Fig. 2). The precision and recall of *A. cerana* were 30.8% and 57.1%, respectively (Table 1). On the other hand, the precision, recall, and F-score of *A. mellifera* were higher than 70% (Table 1). The accuracy of species identification for *A. cerana* was low but *A. mellifera* was high because *A. mellifera* can be observed more easily than *A. cerana* in Japan, especially in urban habitats.

**Table 1:** Precision, recall, and F-score of species identification by biologists. Precision is the number of correct predictions as a certain class divided by the number of all predictions as the class returned by biologists. Recall, which is equivalent to sensitivity, is the number of correct predictions as a certain class divided by the number of test datasets as the class. F-score is the harmonic average of the precision and recall, (2 × precision × recall)/(precision + recall).

Genus	Subgenus	Species	Precision (%)	Recall (%)	F-score (%)
*Apis*	*Apis*	*Apis cerana*	30.8	57.1	40.0
		*A. mellifera*	83.9	74.3	78.8
*Bombus*	*Megabombus*	*Bombus consobrinus*	26.7	28.6	27.6
		*B. diversus*	80.5	58.8	67.9
		*B. ussurensis*	12.5	16.7	14.3
	*Thoracobombus*	*B. pseudobaicalensis* and *B. deuteronymus*	34.8	53.3	42.1
		*B. honshuensis*	34.3	45.8	39.3
	*Pyrobombus*	*B. ardens*	72.4	44.2	54.9
		*B. beaticola*	16.0	21.1	18.2
	*Bombus*	*B. hypocrita*	64.3	65.5	64.9
		*B. ignitus*	37.7	57.1	45.5
		*B. terrestris*	90.0	64.3	75.0

The precisions of common bumblebee species *B. diversus* and *B. ardens* were higher than 70% (Table 1). However, even for common species, intraspecific variation made it difficult to identify species and resulted in 58.8% recall of *B. diversus* and 44.2% recall of *B. ardens* (Table 1). The major hair color of *B. diversus* was orange, but their hair becomes yellowish in cold habitats such as high elevation regions and the northern island Hokkaido. *B. ardens* has not only interspecific similarity but also intraspecific color differences depending on sex (Fig. 1d,f), phenotypic variation, and subspecies (*B. ardens ardens*, *B. ardens sakagamii*, and *B. ardens tsushimanus*), which led to misidentification as other species.

In addition to misidentification due to interspecific similarity and intraspecific variation, there is a tendency to misidentify species when the biologists have not frequently seen them outdoors. The precisions, recalls, and F-scores of *B. consobrinus*, *B. ussurensis*, and *B. beaticola* were lower than 30% (Table 1). These bumble bee species inhabit limited areas or high elevation regions, and they cannot be observed easily in Japan.

### Species identification in species class experiment by Xception

**Figure 3:**
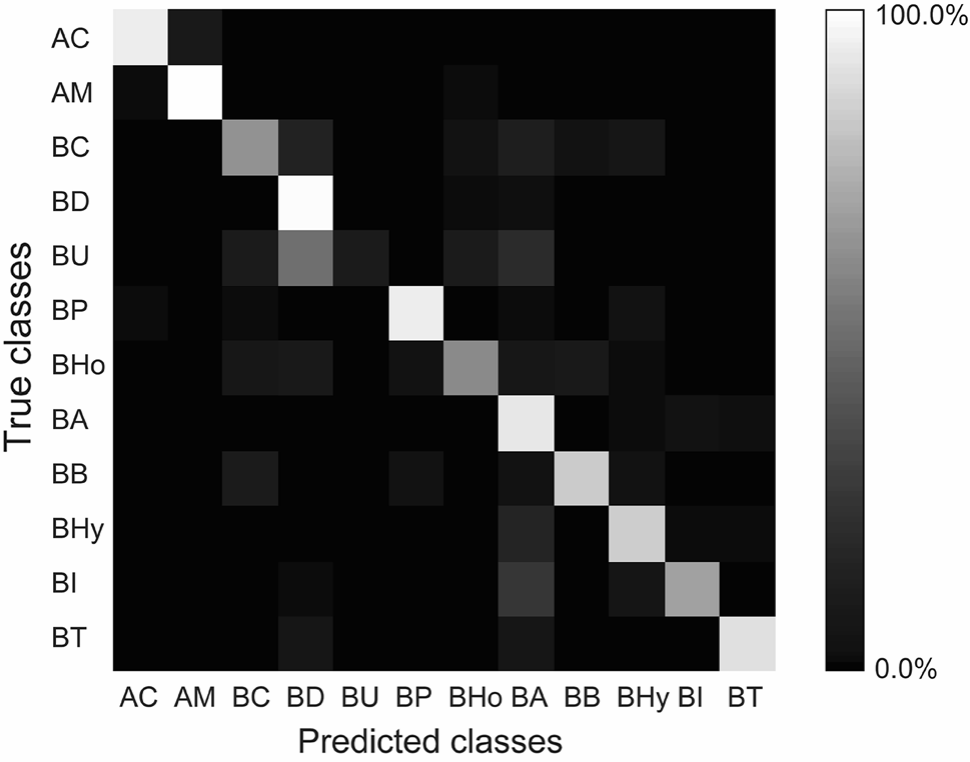
Confusion matrix in species class experiment by Xception. Bright color indicates high percentage of predicted classes for a true class. AC: *Apis cerana*, AM: *A. mellifera*, BC: *Bombus consobrinus*, BD: *B. diversus*, BU: *B. ussurensis*, BP: *B. pseudobaicalensis* and *B. deuteronymus*, BHo: *B. honshuensis*, BA: *B. ardens*, BB: *B. beaticola*, BHy: *B. hypocrita*, BI: *B. ignitus*, and BT: *B. terrestris*.

To compare the accuracy of species identification by deep learning with that by biologists, we categorized bee photographs into different classes according to species in species class experiment. The total accuracy of species identification by Xception reached 83.4% vs. expert’s identification in species class experiment. With exception of precision of *B. terrestris* and recall of *B. ussurensis*, precisions, recalls, and F-scores were higher than those by biologists (Tables 1 and 2). However, the effect of interspecific similarity between female *B. ardens ardens* and female *B. ignitus* was large even in species class experiment by Xception (Fig. 3).

**Table 2:** Precision, recall, and F-score of species identification by Xception in species class experiment. Precision is the number of correct predictions as a certain class divided by the number of all predictions as the class returned by Xception. Recall, which is equivalent to sensitivity, is the number of correct predictions as a certain class divided by the number of test datasets as the class. F-score is the harmonic average of the precision and recall, (2 × precision × recall)/(precision + recall).

Genus	Subgenus	Species	Precision (%)	Recall (%)	F-score (%)
*Apis*	*Apis*	*Apis cerana*	93.5	90.6	92.1
		*A. mellifera*	94.5	96.3	95.4
*Bombus*	*Megabombus*	*Bombus consobrinus*	65.6	61.8	63.6
		*B. diversus*	89.8	94.9	92.3
		*B. ussurensis*	50.0	10.0	16.7
	*Thoracobombus*	*B. pseudobaicalensis* and *B. deuteronymus*	92.5	90.7	91.6
		*B. honshuensis*	86.1	59.6	70.5
	*Pyrobombus*	*B. ardens*	75.4	89.2	81.7
		*B. beaticola*	77.4	80.0	78.7
	*Bombus*	*B. hypocrita*	83.3	81.3	82.3
		*B. ignitus*	84.8	67.2	75.0
		*B. terrestris*	83.9	86.7	85.2

The precisions, recalls, and F-scores of *B. consobrinus* and *B. ussurensis* were lower than 70% in species class experiment by Xception (Table 2). *B. consobrinus* and *B. ussurensis* were sometimes predicted as *B. diversus* (Fig. 3). *B. consobrinus* and *B. ussurensis* were similar to *B. diversus* in respect of body shape because they belong to the same subgenus *Megabombus*. The hair color of *B. consobrinus* (yellow thorax with red band) was similar to that of *B. diversus* (orange thorax), but the hair color of *B. ussurensis* (olive yellow thorax) seemed to be different from that of *B. diversus*. In other misidentification cases, *B. consobrinus* and *B. ussurensis* were sometimes predicted as *B. ardens* (Fig. 3). *B. consobrinus* and *B. ussurensis* were very different from female *B. ardens ardens* (Fig. 1d), but relatively similar to that of male *B. ardens* (Fig. 1f) in the coloration.

### Species identification in color class experiment by Xception

To investigate the effect of intraspecific variation on the accuracy of species identification by deep learning, we categorized bee photographs into different classes according to intraspecific color differences in color class experiment. The total accuracy of species identification by Xception increased to 84.7% vs. expert’s identification in color class experiment. Besides total accuracy, all precisions, recalls, and F-scores were higher than those of biologists (Tables 1 and 3). The recalls of male *B. hypocrita* and male *B. ignitus* became equal to or higher than 80% (Table 3). When weighting the recalls of females and males using sex ratio in test data, the recalls of *B. hypocrita* and *B. ignitus* in color class experiment were 84.1%, and 68.9%, respectively. These were higher than those of species class experiment (*B. hypocrita*: 84.1% in color class vs. 81.3% in species class, *B. ignitus*: 68.9% in color class vs. 67.2% in species class).

**Table 3:** Precision, recall, and F-score of species identification by Xception in color class experiment. F/M within a parenthesis represents female/male. Precision is the number of correct predictions as a certain class divided by the number of all predictions as the class returned by Xception. Recall, which is equivalent to sensitivity, is the number of correct predictions as a certain class divided by the number of test datasets as the class. F-score is the harmonic average of the precision and recall, (2 × precision × recall)/(precision + recall).

Genus	Subgenus	Species	Precision (%)	Recall (%)	F-score (%)
*Apis*	*Apis*	*Apis cerana*	93.8	93.8	93.8
		*A. mellifera*	92.9	96.3	94.5
*Bombus*	*Megabombus*	*Bombus consobrinus*	96.0	70.6	81.4
		*B. diversus*	92.2	94.3	93.3
		*B. ussurensis*	80.0	80.0	80.0
	*Thoracobombus*	*B. pseudobaicalensis* and *B. deuteronymus*	90.6	88.9	89.7
		*B. honshuensis* (F)	82.9	65.9	73.4
	*Pyrobombus*	*B. ardens ardens* (F)	82.5	95.2	88.4
		*B. ardens* (M)	72.4	80.8	76.4
		*B. beaticola* (F)	66.7	78.6	72.1
	*Bombus*	*B. hypocrita* (F)	90.4	81.0	85.5
		*B. hypocrita* (M)	75.9	91.7	83.0
		*B. ignitus* (F)	80.0	66.7	72.7
		*B. ignitus* (M)	88.9	80.0	84.2
		*B. terrestris*	96.2	83.3	89.3

**Figure 4:**
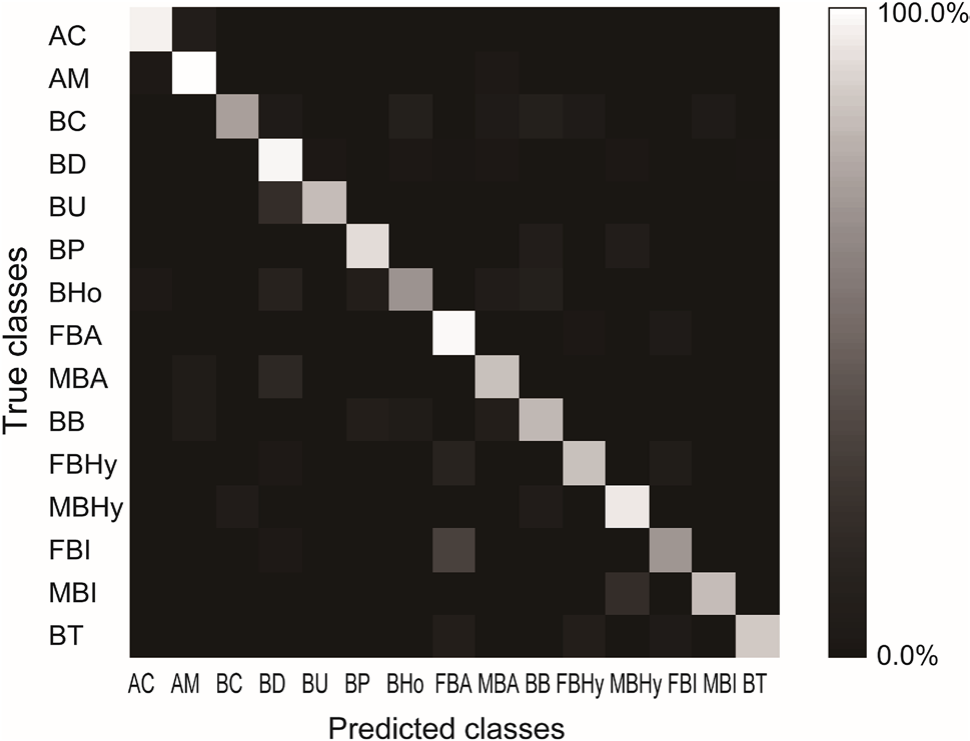
Confusion matrix in color class experiment by Xception. Bright color indicates high percentage. AC: *Apis cerana*, AM: *A. mellifera*, BC: *Bombus consobrinus*, BD: *B. diversus*, BU: *B. ussurensis*, BP: *B. pseudobaicalensis* and *B. deuteronymus*, BHo: *B. honshuensis*, BA: *B. ardens*, BB: *B. beaticola*, BHy: *B. hypocrita*, BI: *B. ignitus*, and BT: *B. terrestris*. F and M in front of them mean female and male, respectively.

The precisions, recalls and F-scores of *B. consobrinus* and *B. ussurensis* were higher than 70% in color class experiment (Table 3), even though their classes were the same as in species class experiment. A part of the reason was the decrease in misidentification cases as male *B. ardens* (Fig. 4, Tables S2 and S3 in Appendix S2 in Supplementary information). In species class experiment by Xception, *B. consobrinus* was predicted as *B. ardens* in 30.8% of misidentification cases, and *B. ussurensis* was predicted as *B. ardens* in 22.2% of misidentification cases (Table S2 in Appendix S2 in Supplementary information). In color class experiment, *B. consobrinus* was predicted as male *B. ardens* in only 10.0% of misidentification cases, and *B. ussurensis* was not predicted as male *B. ardens* (Table S3 in Appendix S2 in Supplementary information). Both of the negative effects of intraspecific variation and interspecific similarity were mitigated in color class experiment.

## Discussion

We considered that species identification by deep learning can increase the availability of organism photographs taken by citizens. In this study, a deep convolutional neural network, Xception, trained bee species identification using the photograph dataset. The total accuracy of species identification by Xception was evaluated by comparing the total accuracy of species identification by biologists. 50 biologists with different levels of knowledge tested species identification test of nine bee photographs selected randomly from the photograph dataset. The total accuracy of species identification by Xception (83.4% and 84.7%) was much higher than that of biologists (53.7%). Deep convolutional neural networks (DCNNs) will be able to identify species more accurately than most participants in citizen science programs by learning numerous photographs. In addition, if participants have a lot of photographs to identify species or if the experts have no time to identify species, DCNNs will be able to identify species faster than the experts. The rapid response of species identification from DCNNs will provide a benefit for participants, such as increasing the efficiency of species identification training and the availability of their data before experts’ verification. Although some researchers may think that the accuracy of species identification by DCNNs is not high enough to use for scientific researches, they can use results of species identification in scientific researches considering the error rate of species identification. Statistics for erroneous data (e.g.^24^) can be applied to species identification data with errors. Otherwise, the results of species identification in collaboration with DCNNs and experts can be used for scientific researches. DCNNs can output the certainty of species identification numerically. Experts can select photographs with low certainty of species identification by DCNNs, and judge whether they should identify species in the photographs or eliminate the photographs from data.

The total accuracy of species identification by biologists was low (53.7%), but comparable to the accuracy of species identification by experts and non-experts using field guides^25^, or slightly higher than those of participants in other citizen science programs^14^. The experimental conditions of these previous studies were greatly different: experts and non-experts answered whether two bumble bee images from different field guides were the same species^25^, and participants in citizen science programs answered the species of bumble bee photographs^14^. Although the experimental conditions were different among previous studies and this study, all results would support the difficulty in identifying bee species. The interspecific similarity of bee species hindered species identification by biologists. As we expected, the negative effect of interspecific similarity (Müllerian mimicry) between female *B. ardens ardens* (black body with orange tail in Fig. 1d) and female *B. ignitus* (black body with orange tail in Fig. 1e) was strong for biologists (Fig. 2). The intraspecific variation of bee species also hindered species identification by biologists. Low recalls of *B. diversus* and *B. ardens* in species identification by biologists were partly attributed to the effect of intraspecific variation.

The negative effect of interspecific similarity between female *B. ardens ardens* and *B. ignitus* was large even in species identification by DCNN Xception (Figs. 3 and 4, Tables S2 and S3 in Appendix S2 in Supplementary information), though the recalls of female *B. ardens* and *B. ignitus* were much higher than those by biologists (Tables 1, 2, and 3). Previous studies of fine-grained image classification by deep learning reported the negative effect of interspecific similarity in the same genus on the accuracy of species identification. In study of 200 bird species identification, five of the six cases that are most confused with each other were species in the same genus^26^. We considered that the total accuracy of species identification in species class experiment by Xception was relatively high because our target was a small number of species in a small number of genera in the same family. In an extreme case, iNaturalist photograph dataset consisted of over 5000 species in both animals and plants, and the accuracy of species identification by deep learning was 67%^27^. When DCNNs learn a large number of species across many genera or families, differences between similar species in the same genus may be too small to learn more.

The negative effect of intraspecific variation was also large in species class experiment, but mitigated in color class experiment by Xception. Focusing on species with sexual dimorphism, the recalls of *B. hypocrita* and *B. ignitus* in color class experiment became higher than those of species class experiment by Xception. Interestingly, color class experiment improved the identification of species with interspecific similarity as well as species with intraspecific variation. Intraspecific variation was not independent of interspecific similarity: one type of hair colors in intraspecific variation was similar to the hair color of other species. By categorizing color differences of intraspecific variation into different color classes, Xception learned intraspecific variation efficiently, and distinguished one type of hair colors in the species from the hair color of other species. In color class experiment, Xception could learn male *B. ardens,* which has different hair color from female *B. ardens*. Then, Xception distinguished male *B. ardens* from *B. consobrinus* and *B. ussurensis* more precisely in color class experiment than species class experiment (Table 3 and Fig. 4, Appendix S2 in Supplementary information).

In general, the accuracy of species identification does not always increase by detailed classification because there is a trade-off between data quality and quantity, or the shortage of volume for minor species/variants. Species present in organism photographs are usually imbalanced^8,15^, and categorization into more classes will increase the imbalance in the number of photographs per class and worsen the shortage of photographs for specific classes. This shortage reduces the accuracy of species identification for the specific classes, and may lower the total accuracy of species identification. In this study, species identification for some minor classes (less than 40 original photographs) had to be abandoned in color class experiment, but data augmentation mitigated the negative effect of photograph shortage for the other classes. By categorizing color differences of intraspecific variation into different classes, the total accuracy of species identification by Xception increased from 83.4% to 84.7% in color class experiment. If we did not normalize it (see “The accuracy of species identification” subsection in M&M), the accuracy of species identification in color class experiment reached 86.7%. In future works, we will be able to improve the accuracy of identifying species in minor classes to incorporate other methods like a linear support vector machine^28^.

These photographs and species identification can be used for scientific researches such as investigating native species distributions, conserving habitats of rare species, and detecting invasion of alien species. In this study, the proportion of species in test data reflects the proportion of species in training data, and deep learning was conducted to maximize the accuracy of species identification. In that case, the recall of common species is high, but the recall of rare species may become low. If the major purpose is to detect rare species or uncommon alien species, it will be ideal to conduct deep learning so as to mitigate the imbalance of species in training data (e.g., the constant volume of photographs per class) or maximize the recall average. Depending on the purpose, we must select a methodology for increasing the availability and reliability of photographs taken by citizens.

## Materials and methods

### Citizen science program “*Hanamaru-maruhana* national census”

We asked citizens to take bee photographs and send them by e-mails in citizen science program “*Hanamaru-Maruhana* national census (Bumble bee national census in English)” (http://hanamaruproject.s1009.xrea.com/hanamaru_project/index_E.html)^8^. We gave citizens previous notice that their photographs were going to be used for scientific studies, and for other non-profit activities on our homepage and flyers. From 2013 to 2016, we collected roughly 5000 photographs taken by citizens. Citizens sent photographs of various bee species, but most of them were bumble bees and honey bees. They have interspecific similarity and intraspecific variation, making it difficult for non-experts to identify species. Since species identification was not a requirement for participants, most citizens sent bee photographs without species identification. These bees were identified by one of the authors, J. Yokoyama. These bees are relatively easy for experts to identify because only two honey bee species and 16 bumble bee species inhabit the Japanese archipelago excluding the Kurile Islands. The consistency of species identification by J. Yokoyama was 95% for 15 bumble bee species, and 97.7% for major six bumble bee species in our test using 100 bumble bee photographs^8^.

### Bee photographs used for deep learning

From bee species observed in citizen science program “*Hanamaru-maruhana* national census (Bumble bee national census in English)”, we selected two honey bee species and 10 bumble bee species having interspecific similarity and intraspecific variation. Two honey bee species consisted of *Apis cerana* Fabricius, and *A. mellifera* Linnaeus. 10 bumble bee species consisted of *Bombus consobrinus* Dahlbom, *B. diversus* Smith, *B. ussurensis* Radoszkowski, *B. pseudobaicalensis* Vogt, *B. honshuensis* Tkalcu, *B. ardens* Smith, *B. beaticola* Tkalcu, *B. hypocrita* Perez, *B. ignitus* Smith, and *B. terrestris* Linnaeus. To increase training data of *B. pseudobaicalensis*, we added photographs of *B. deuteronymus* Schulz to photographs of *B. pseudobaicalensis* because they can rarely be distinguished using only photographic images (see http://hanamaruproject.s1009.xrea.com/hanamaru_project/identification_E.html for the details of their color patterns). We primarily used photographs taken by citizens from 2013 to 2015 in the citizen science program, but also used photographs taken by citizens in 2016 if the number of photographs for a certain class was small.

We cropped a bee part as a rectangle image from a photograph to reduce background effects. We increased the number of photographs by data augmentation (Fig. S1 in Appendix S1 in Supplementary information). Please see Appendix S1 in Supplementary information for the details of “Data augmentation.” We assigned 70, 10, and 20% of the total data of the training dataset, validation dataset, and test dataset, respectively. Please see Appendix S1 in Supplementary information for the details of “Data split and training parameters”.

### Deep convolutional neural network (DCNN)

In this study, we chose a deep convolutional neural network Xception, as it provides a good balance between the accuracy of the model on one hand and a smaller network size on the other. We adopted transfer learning^21,22^ and data augmentation^23^ to solve the issue of a shortage of photographs. The Xception network has a depth of 126 layers (including activation layers, normalization layers etc.) out of which 36 are convolution layers. In this study, we employed the pretrained Xception V1 model provided on the Keras homepage. Please see Appendix S1 in Supplementary information for the details of “Xception”, and “Transfer learning.” For the training, we chose a learning rate of 0.0001 and a momentum of 0.9.

### Species identification by biologists

We asked 50 biologists to identify the species present in nine photographs selected randomly from the photograph dataset using a questionnaire form. Their professions were forth undergraduate student (16%), Master’s student (14%), Ph.D. student (12%), Postdoctoral fellow (26%), Assistant professor (6%), Associate professor (12%), Professors (6%), and others (8%). Their research organisms were honey bees (6%), bumble bees (14%), bees (6%), insects (12%), plants and insects (12%), plants (22%), and others such as fishes, reptiles, and mammals (28%). 14% of the biologists were studying bumble bees, but they did not need to identify all bumble bee species in their researches because only several species inhabit their study areas. We allowed the biologists to see field guide books, illustrated books, and websites. We did not limit the method or time to identify the species of photographs to simulate the species identification of actual citizen science programs as much as possible, except for asking experts. The experiment was approved by the Ethics Committee in Tohoku University, and carried out in accordance with its regulations. Informed consent was obtained from the biologists.

### Species identification in species class experiment by Xception

We conducted species class experiment by categorizing photographs into different classes according to species. A total of 3779 original photographs were used in species class experiment (Table S1 in Appendix S1 in Supplementary information). These photographs were classified into 12 classes according to species. We inputted test dataset to Xception, and recorded their predicted classes.

### Species identification in color class experiment by Xception

We conducted color class experiment by categorizing photographs into different classes according to intraspecific color differences. Photographs of *B. ardens* were classified into the following four classes: female *B. ardens ardens*, *B. ardens sakagamii*, *B. ardens tsushimanus*, and male *B. ardens* (Table S1 in Appendix S1 in Supplementary information). Photographs of *B. honshuensis*, *B. beaticola*, *B. hypocrita*, and *B. ignitus* were classified into female and male classes. In trial experiments, we had found that the Xception cannot learn images in minor classes if the number of original photographs in the classes was less than 40. No photographs in the class were predicted correctly, and no photographs in the other classes were predicted as the class. Therefore, in color class experiment, we did not use the photographs of minor classes (*B. ardens* subspecies: *B. ardens sakagamii* and *B. ardens tsushimanus*, male *B. honshuensis*, and male *B. beaticola*). Therefore, a total of 3681 original photographs were used in color class experiment (Table S1 in Appendix S1 in Supplementary information). They were classified into 15 classes according to intraspecific color differences in addition to species classes. We inputted test dataset to Xception, and recorded their predicted classes. To compare the total accuracy of color class experiment by Xception with those of other experiments, it was normalized using the number of test data including those of the minor classes, assuming that all test data of the minor classes were misidentified.

### The accuracy of species identification

We calculated total accuracy, precision, recall, and F-score in each class. Total accuracy is the number of total correct predictions divided by the number of all test datasets. Note that the total accuracy of color class experiment by Xception was normalized using the number of test data including those of the minor classes. It reduces the total accuracy of color class experiment by Xception, and enables to compare with those by biologists and species class experiment by Xception directly. Precision is the number of correct predictions as a certain class divided by the number of all predictions as the class returned by biologists or Xception. Recall, which is equivalent to sensitivity, is the number of correct predictions as a certain class divided by the number of test datasets as the class. F-score is the harmonic average of the precision and recall, (2 × precision × recall)/(precision + recall).

To show the effect of interspecific similarity on the accuracy of species identification, we used confusion matrix. The confusion matrix represents the relationship between true and predicted classes. Each row indicates the proportion of predicted classes in a true class. All correct predictions are located in the diagonal of the matrix, wrong predictions are located out of the diagonal. In species identification by biologists, “Others” class represents cases that they wrote no species name or a species name other than two honey bee species and 10 bumble bee species in the answer column.

## Supplementary Information

Supplementary Information.
