# Supplementary material for: Deep learning increases the availability of organism photographs taken by citizens in citizen science programs
Source: Sci Rep. 2022 Jan 24;12:1210. doi: 10.1038/s41598-022-05163-5 (PMC8786926; doi:10.1038/s41598-022-05163-5)
Supplement: Supplementary file 1 — Supplementary Information. [file 41598_2022_5163_MOESM1_ESM.docx]

Supplementary Information

Deep learning increases the availability of organism photographs taken by citizens in citizen science programs

Yukari Suzuki-Ohno, Thomas Westfechtel, Jun Yokoyama, Kazunori Ohno, Tohru Nakashizuka, Masakado Kawata, and Takayuki Okatani

Appendix S1. Xception, Transfer learning, Data augmentation, Data split and training parameters

Xception

Since the introduction of AlexNet (Krizhevsky et al. 2012), DCNNs have gained a lot of popularity mainly due to their huge success in image classification, object detection and many other areas of computer vision. In the subsequent years, researchers increased the performance by increasing the depth of the network with ZF net in 2013 (Zeller and Fergus 2014) and VGG net (Simonyan and Zisserman 2014). The performance was further increased through the introduction of residual connections in the ResNet (He et al. 2015) architecture allowing for training even deeper networks. ResNet also achieved to achieve a lower classification error than humans in the ILSVRC challenge were pictures have to be assigned to one of 1000 classes.

Beside the trend of steadily developing deeper networks, the inception v1 or GoogLeNet network (Szegedy et al. 2015) introduced factoring convolutions split into multiple branches operating successively on channels and then on space. Xception network (Chollet et al. 2017) is inspired by multiple branches of the Inception network and extended this approach with depthwise separable convolution. At the same time, it employs residual connections between the convolution layer from the ResNet architecture. In this study, we used the Xception network that has a depth of 126 layers (including activation layers, normalization layers etc.) out of which 36 are convolution layers. The architecture is a linear stack of depthwise separable convolution layers with residual connections. The model consists a total of 22,910,480 parameters.

Transfer learning

Transfer learning takes the weights of the network trained on a large dataset and transfers the weights to initialize a network for a classification task for a different dataset (most times smaller dataset). This weight transfer, and thereby the transfer of the learned feature representations, is followed by a finetuning on the new dataset. This transfer learning process helps with the generalization and increases the accuracy especially in case of few training data (Oquab et al. 2014; Yosinski et al. 2014). As this practice is common use, researchers publish the parameters of convolutional networks online, mostly pretrained on the ImageNet dataset. In this study, we employed the pretrained Xception V1 model provided on the Keras homepage, which achieves a top-1 validation accuracy of 0.790 and a top-5 validation accuracy of 0.945 on the ImageNet dataset.

Data augmentation

As we used a small dataset, we chose to augment the training data to generate further input data. The pictures in the dataset were originally not square; the height and width of each picture was different. However, convolutional neural networks (CNNs) require square inputs, with the Xception requiring pictures with a resolution of 299 x 299 as input. Directly resizing the pictures to this resolution would cause the pictures to be stretched, thus altering the body proportions of the bees. In order to avoid this, we sampled the picture as square so that the smaller dimension of the original picture has a size of 299 pixels. We simultaneously increased the size of the dataset by data augmentation. If the height of the picture was larger than the width, the top third of the image was sampled, followed by the middle third of the image, and finally the bottom third of the image was samples. If the height of the picture was smaller than the width, the left, middle, and right sections of the image were sampled. All three images were set as training data. To further increase the number of training data, we augmented the pictures in the following four ways:

1. Flip the image horizontally.

2. Zoom up to *x* into the image.

3. Shifted the width/height for up to *x*.

4. Rotated to image for up to *x*°.

Fig. S1 shows this process for one original image (Fig. S1(a)). Other data augmentation methods were proposed in the field of computer science, but traditional data augmentation described above was one of successful data augmentation methods (Perez and Wang 2017).

We increased the number of images by at least 6 times through 3 samples (top, middle, bottom) and horizontally flipping each of the samples (Fig. S1(b)-(g)). The zoom, shift, and rotation operations were done on the fly: Each time one of the images was used in the training these operations were executed with new random values (random within the defined range), resulting in a unique image each time (Fig. S1(h)-(j)). After evaluating different values of *x*, we determined *x* as 30.

In this study, we did not use cut-out (DeVries and Taylor 2017) or random erasing method (Zhong et al. 2017) in data augmentation. These methods may be not so effective because bees are often partially invisible in our original photos.

Data split and training parameters

We split the whole original photos (Table S1) into training, validation, and test datasets. The CNN was finetuned on the training dataset and was validated on the validation dataset. Training continued until ten consecutive steps do not improve the accuracy on the validation dataset. We checked the effects of the proportion of training and validation datasets on the accuracy of species identifications in trial experiments. Based on these results, we assigned 70, 10, and 20% of the total data for the training dataset, validation dataset, and test dataset, respectively.

For the training, we chose a learning rate of 0.0001 and a momentum of 0.9. The training is continued until ten consecutive steps did not improve the accuracy on the validation dataset. During the testing, we inputted the middle section of the image and their horizontally flipped versions (Fig. S1). We chose the final result as the maximum score of the sum of the single scores for each inputted image.

References

DeVries, T. and Taylor, G.W. (2017) Improved regularization of convolutional neural networks with cutout. arXiv:1708.04552

He, K., Zhang, X., Ren, S., and Sun, J. (2016) Deep residual learning for image recognition. Proceedings of the IEEE conference on computer vision and pattern recognition, 770-778.

Krizhevsky, A., Sutskever, I., and Hinton, G.E. (2012) Imagenet classification with deep convolutional neural networks. Advances in neural information processing systems, 1097-1105.

Simonyan, K., and Zisserman, A. (2014) Very deep convolutional networks for large-scale image recognition. arXiv preprint arXiv:1409.1556.

Szegedy, C., Liu, W., Jia, Y., Sermanet, P., Reed, S., Anguelov, D., Erhan, D., Vanhoucke, V., and Rabinovich, A. (2015) Going deeper with convolutions. Proceedings of the IEEE conference on computer vision and pattern recognition, 1-9.

Zeiler, M.D., and Fergus, R. (2014) Visualizing and understanding convolutional networks." In European conference on computer vision, pp. 818-833. springer, Cham.

Zhong, Z., Zheng, L., Kang, G., Li, S., and Yang, Y. (2017) Random erasing data augmentation. arXiv:1708.04896

Table S1. The number of original bee photographs before data augmentation. F and M within a parenthesis represents female and male, respectively. The number within a parenthesis means that we separated photographs of *Bombus ardens sakagamii*, *B. ardens tsushimanus*, male *B. beaticola*, and male *B. honshuensis*, but did not use them because the number of the photographs was too small to learn in color class experiment.

| Subgenus | Species | Species class | Color class |
| --- | --- | --- | --- |
| *Apis* | *Apis cerana* | 154 | 154 |
|  | *A. mellifera* | 268 | 268 |
| *Megabombus* | *Bombus consobrinus* | 160 | 160 |
|  | *B. diversus* | 872 | 872 |
|  | *B. ussurensis* | 48 | 48 |
| *Thoracobombus* | *B. pseudobaicalensis* and  *B. deuteronymus* | 268 | 268 |
|  | *B. honshuensis* | 253 |  |
|  | *B. honshuensis* (F) |  | 216 |
|  | *B. honshuensis* (M) |  | (37) |
| *Pyrobombus* | *B. ardens* | 784 | - |
|  | *B. ardens ardens* (F) | - | 610 |
|  | *B. ardens sakagamii* (F) | - | (17) |
|  | *B. ardens tsushimanus* (F) | - | (30) |
|  | *B. ardens* (M) | - | 127 |
|  | *B. beaticola* | 147 | - |
|  | *B. beaticola* (F) | - | 133 |
|  | *B. beaticola* (M) | - | (14) |
| *Bombus* | *B. hypocrita* | 397 | - |
|  | *B. hypocrita* (F) | - | 282 |
|  | *B. hypocrita* (M) | - | 115 |
|  | *B. ignitus* | 281 | - |
|  | *B. ignitus* (F) | - | 236 |
|  | *B. ignitus* (M) | - | 45 |
|  | *B. terrestris* | 140 | 140 |
| Photographs |  | 3779 | 3681 (98) |
| Classes |  | 12 | 15 |

Fig. S1. (a) Original image, (b) Top, (c) Middle, (d) Bottom, (e)-(g) Flipped versions, (h)-(j) Examples of randomly zoomed, shifted, and rotated versions using middle image.


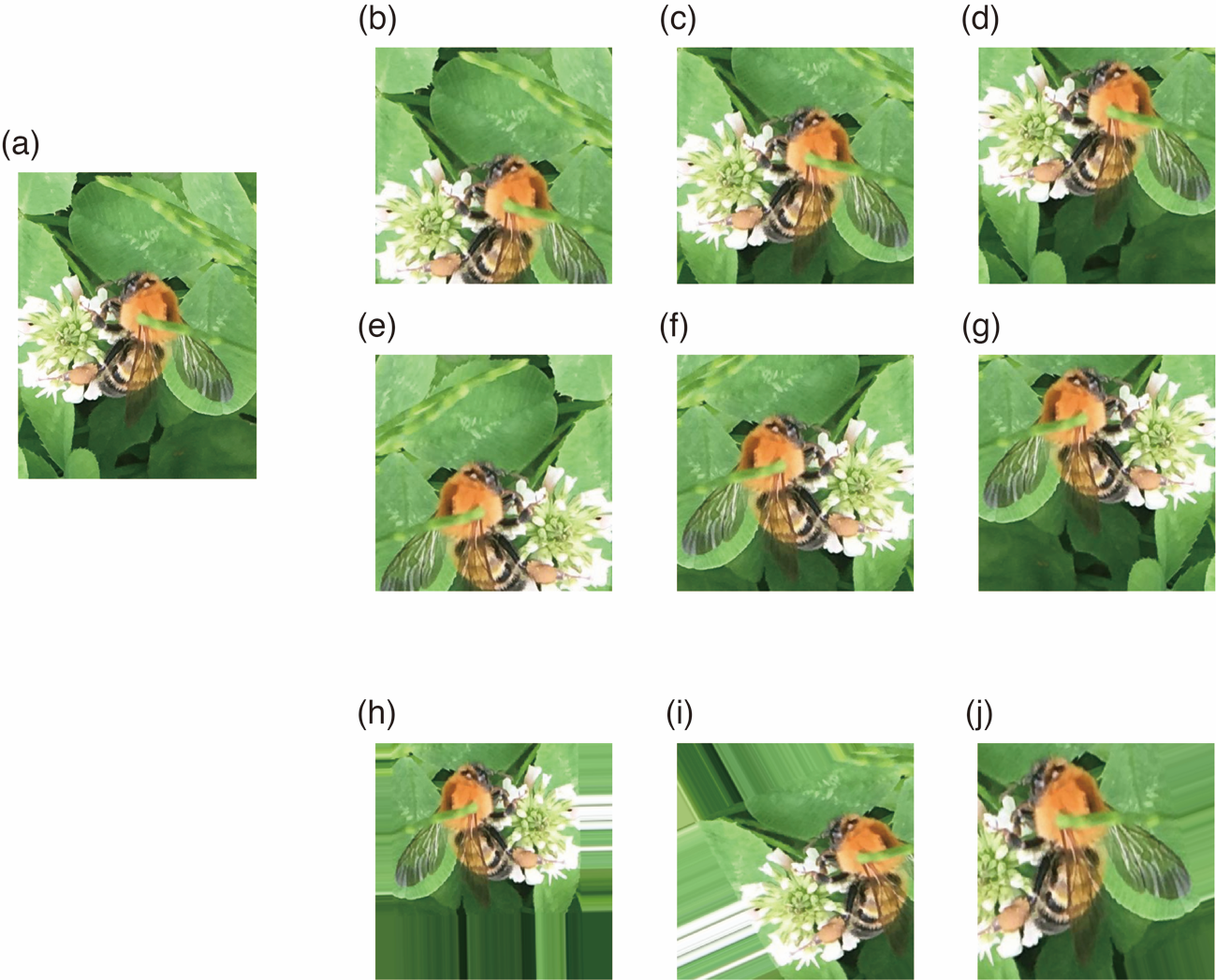


Appendix S2. Patterns of misidentification cases

When misidentification cases were rare, it is difficult to find which species this species was misidentified as by seeing a confusion matrix. Therefore, we investigated the percentage of predicted species among misidentification cases.

In species class experiment, the case where female *B. ignitus* was predicted as *B. ardens* corresponded to 100.0% of misidentification cases of female *B. ignitus* (percentages in parentheses for BI in Table S2). The case where female *B. ardens ardens* was predicted as *B. ignitus* corresponded to 54.5% of misidentification cases of female *B. ardens ardens*. The case where *B. consobrinus* was predicted as *B. ardens* was 30.8% of misidentification cases, and the case where *B. ussurensis* was predicted as *B. ardens* was 22.2% of misidentification cases (Table S2).

In color class experiment, the case where female *B. ignitus* was predicted as female *B. ardens ardens* corresponded to 93.8% of misidentification cases of female *B. ignitus*. Conversely, the case where female *B. ardens ardens* was predicted as female *B. ignitus* corresponded to 66.7% of misidentification cases of female *B. ardens* (Table S3). The cases where *B. consobrinus* or *B. ussurensis* was predicted as male *B. ardens* became rare in color class experiment. *B. consobrinus* was predicted as male *B. ardens* in 10.0% of misidentification cases, and *B. ussurensis* was not predicted as male *B. ardens* in color class experiment (Table S3).

Table S2. Patterns of misidentification cases in species class experiment. Columns are true and rows are predicted species in misidentification cases. AC: *Apis cerana*, AM: *A. mellifera*, BC: *Bombus consobrinus*, BD: *B. diversus*, BU: *B. ussurensis*, BP: *B. pseudobaicalensis* and *B. deuteronymus*, BHo: *B. honshuensis*, BA: *B. ardens*, BB: *B. beaticola*, BHy: *B. hypocrita*, BI: *B. ignitus*, BT: *B. terrestris*. Percentages in parentheses in BA, BHy, and BI represent the patterns in test data of female photos*.

* Percentages in parentheses in BA represent those of female *B. ardens ardens*.

|  | AC | AM | BC | BD | BU | BP | BHo | BA | BB | BHy | BI | BT |
| --- | --- | --- | --- | --- | --- | --- | --- | --- | --- | --- | --- | --- |
| AC | - | 100.0 | 0.0 | 0.0 | 0.0 | 0.0 | 0.0 | 0.0 | 0.0 | 0.0 | 0.0 | 0.0 |
| AM | 50.0 | - | 0.0 | 0.0 | 0.0 | 0.0 | 50.0 | 0.0 | 0.0 | 0.0 | 0.0 | 0.0 |
| BC | 0.0 | 0.0 | - | 38.5 | 0.0 | 0.0 | 7.7 | 30.8 | 7.7 | 15.4 | 0.0 | 0.0 |
| BD | 0.0 | 0.0 | 11.1 | - | 11.1 | 0.0 | 22.2 | 44.4 | 0.0 | 11.1 | 0.0 | 0.0 |
| BU | 0.0 | 0.0 | 11.1 | 55.6 | - | 0.0 | 11.1 | 22.2 | 0.0 | 0.0 | 0.0 | 0.0 |
| BP | 20.0 | 0.0 | 20.0 | 0.0 | 0.0 | - | 0.0 | 20.0 | 0.0 | 40.0 | 0.0 | 0.0 |
| BHo | 0.0 | 0.0 | 19.0 | 23.8 | 0.0 | 9.5 | - | 19.0 | 23.8 | 4.8 | 0.0 | 0.0 |
| BA | 0.0 | 0.0 | 5.9 | 5.9 | 0.0 | 5.9 | 0.0 | - | 5.9 | 17.6 | 35.3 | 23.5 |
|  |  |  | (0.0) | (0.0) |  | (0.0) |  |  | (0.0) | (18.2) | (54.5) | (27.3) |
| BB | 0.0 | 0.0 | 50.0 | 0.0 | 0.0 | 16.7 | 0.0 | 16.7 | - | 16.7 | 0.0 | 0.0 |
| BHy | 0.0 | 0.0 | 0.0 | 0.0 | 0.0 | 0.0 | 0.0 | 86.7 (92.3) | 0.0 | - | 6.7 (0.0) | 6.7 (7.7) |
| BI | 0.0 | 0.0 | 0.0 | 5.3 (0.0) | 0.0 | 0.0 | 0.0 | 73.7 (100) | 0.0 | 15.8 (0.0) | - | 0.0 |
| BT | 0.0 | 0.0 | 0.0 | 50.0 | 0.0 | 0.0 | 0.0 | 50.0 | 0.0 | 0.0 | 0.0 | - |

Table S3. Patterns of misidentification cases in color class experiments. Columns are true and rows are predicted species in misidentification cases. AC: *Apis cerana*, AM: *A. mellifera*, BC: *Bombus consobrinus*, BD: *B. diversus*, BU: *B. ussurensis*, BP: *B. pseudobaicalensis* and *B. deuteronymus*, BHo: *B. honshuensis*, FBA: female *B. ardens*, MBA: male *B. ardens*, BB: *B. beaticola*, FBHy: female *B. hypocrita*, MBHy: male *B. hypocrita*, FBI: female *B. ignitus*, MBI: male *B. ignitus*, BT: *B. terrestris*.

|  | AC | AM | BC | BD | BU | BP | BHo | FBA | MBA | BB | FBHy | MBHy | FBI | MBI | BT |
| --- | --- | --- | --- | --- | --- | --- | --- | --- | --- | --- | --- | --- | --- | --- | --- |
| AC | - | 100.0 | 0.0 | 0.0 | 0.0 | 0.0 | 0.0 | 0.0 | 0.0 | 0.0 | 0.0 | 0.0 | 0.0 | 0.0 | 0.0 |
| AM | 50.0 | - | 0.0 | 0.0 | 0.0 | 0.0 | 0.0 | 0.0 | 50.0 | 0.0 | 0.0 | 0.0 | 0.0 | 0.0 | 0.0 |
| BC | 0.0 | 0.0 | - | 10.0 | 0.0 | 0.0 | 30.0 | 0.0 | 10.0 | 30.0 | 10.0 | 0.0 | 0.0 | 10.0 | 0.0 |
| BD | 0.0 | 0.0 | 0.0 | - | 20.0 | 0.0 | 20.0 | 10.0 | 20.0 | 0.0 | 0.0 | 20.0 | 0.0 | 0.0 | 10.0 |
| BU | 0.0 | 0.0 | 0.0 | 100.0 | - | 0.0 | 0.0 | 0.0 | 0.0 | 0.0 | 0.0 | 0.0 | 0.0 | 0.0 | 0.0 |
| BP | 0.0 | 0.0 | 0.0 | 0.0 | 0.0 | - | 0.0 | 0.0 | 0.0 | 50.0 | 0.0 | 50.0 | 0.0 | 0.0 | 0.0 |
| BHo | 6.7 | 0.0 | 0.0 | 33.3 | 0.0 | 20.0 | - | 0.0 | 13.3 | 26.7 | 0.0 | 0.0 | 0.0 | 0.0 | 0.0 |
| FBA | 0.0 | 0.0 | 0.0 | 0.0 | 0.0 | 0.0 | 0.0 | - | 0.0 | 0.0 | 33.3 | 0.0 | 66.7 | 0.0 | 0.0 |
| MBA | 0.0 | 20.0 | 0.0 | 80.0 | 0.0 | 0.0 | 0.0 | 0.0 | - | 0.0 | 0.0 | 0.0 | 0.0 | 0.0 | 0.0 |
| BB | 0.0 | 16.6 | 0.0 | 0.0 | 0.0 | 33.3 | 16.6 | 0.0 | 33.3 | - | 0.0 | 0.0 | 0.0 | 0.0 | 0.0 |
| FBHy | 0.0 | 0.0 | 0.0 | 9.1 | 0.0 | 0.0 | 0.0 | 63.6 | 0.0 | 0.0 | - | 0.0 | 27.3 | 0.0 | 0.0 |
| MBHy | 0.0 | 0.0 | 50.0 | 0.0 | 0.0 | 0.0 | 0.0 | 0.0 | 0.0 | 50.0 | 0.0 | - | 0.0 | 0.0 | 0.0 |
| FBI | 0.0 | 0.0 | 0.0 | 6.3 | 0.0 | 0.0 | 0.0 | 93.8 | 0.0 | 0.0 | 0.0 | 0.0 | - | 0.0 | 0.0 |
| MBI | 0.0 | 0.0 | 0.0 | 0.0 | 0.0 | 0.0 | 0.0 | 0.0 | 0.0 | 0.0 | 0.0 | 100.0 | 0.0 | - | 0.0 |
| BT | 0.0 | 0.0 | 0.0 | 0.0 | 0.0 | 0.0 | 0.0 | 40.0 | 0.0 | 0.0 | 40.0 | 0.0 | 20.0 | 0.0 | - |
